# Molecular genetic characterization of a novel HIV-1 circulating recombinant form (CRF205_0107) among men who have sex with men in Hangzhou, China

**DOI:** 10.3389/fmicb.2026.1794333

**Published:** 2026-04-09

**Authors:** Ling Ye, Ke Xu, Wenjie Luo, Sisheng Wu, Zhou Sun, Xingliang Zhang, Jiafeng Zhang, Qin Fan, Min Zhu

**Affiliations:** 1 Department of HIV/AIDS Control and Prevention, Hangzhou Center for Disease Control and Prevention (Hangzhou Health Supervision Institution), Hangzhou, China; 2 Zhejiang Key Laboratory of Multi-Omics in Infection and Immunity, Hangzhou, China; 3 Department of HIV/AIDS Control and Prevention, Zhejiang Provincial Center for Disease Control and Prevention, Hangzhou, China

**Keywords:** circulating recombinant form, HIV-1, men who have sex with men, molecular epidemiology, near-full-length genome

## Abstract

**Introduction:**

Circulating recombinant forms (CRFs) play a critical role in the global transmission of HIV-1. In China, where CRF01_AE and CRF07_BC are the predominant circulating strains, second-generation recombinant viruses derived from these two CRFs are continually emerging. This study identified a novel CRF derived from CRF01_AE and CRF07_BC, designated CRF205_0107, and analyzed its genomic structure, parental lineages, and coreceptor usage.

**Methods:**

A dataset of HIV-1 *pol* gene sequences was analyzed to screen for suspected unique recombinant clusters in Hangzhou, China. Plasma samples from HIV-1 infected individuals without known epidemiological linkages were selected for in-depth investigation. Near-full-length genomes (NFLGs) were generated using a two-segment amplification strategy. BLAST searches were conducted to detect highly homologous sequences. SimPlot software was used to analyze recombination patterns. Further validation was performed by constructing maximum-likelihood (ML) trees using IQ-TREE, while BEAST was employed to estimate the probable time of origin of the parental strains. Coreceptor tropism was predicted based on V3-loop sequences using the Geno2pheno algorithm.

**Results:**

Four HIV-1 NFLGs were obtained. BLAST analysis revealed that a previously reported NFLG sequence (Accession No. OP921950) from a man who has sex with men (MSM) individual in Hebei Province shared 98% similarity. Phylogenetic analysis demonstrated that these sequences formed a distinct monophyletic clade with 100% bootstrap support. Recombination analysis indicated that all five genomes shared a highly similar recombination pattern. Sub-regional phylogenetic analysis revealed that segments derived from CRF01_AE and CRF07_BC all clustered with the lineage associated with Chinese MSM (CRF01_AE_C4, CRF07_BC_N). Bayesian analysis estimated that CRF205_0107 originated around 2017–2018. Coreceptor tropism analysis based on the V3 loop indicated that all five NFLGs were CXCR4-tropic.

**Discussion:**

The emergence of CRF205_0107 provides further evidence that second-generation HIV-1 recombinants involving CRF01_AE and CRF07_BC are frequently generated in China, reflecting an increasingly complex HIV-1 epidemic among MSM populations. This complexity may further drive the accelerated spread of HIV-1. These findings underscore the importance of strengthening the identification and molecular epidemiological surveillance of CRF01_AE/CRF07_BC recombinant viruses, and highlight the urgency of curbing HIV-1 transmission among MSM in China.

## Introduction

HIV-1 exhibits high genetic diversity and rapid evolution. Through continuous mutation, it can increase its biological fitness, evade host immune responses, and generate drug-resistant variants, posing significant challenges to diagnosis, treatment, and vaccine development. As the primary causative agent of the global AIDS pandemic, HIV-1 group M has diversified into multiple prevalent genetic lineages, including subtypes (ten currently recognized: A–D, F–H, J–L; Subtypes E and I were later found not to be pure subtypes through full-length genome analysis), sub-subtypes (A1–A6, F1, F2), and CRFs (Nájera et al., 2002). According to the Los Alamos HIV Sequence Database (2025), 178 CRFs have been documented to date^1^. The ongoing rise in CRFs is driven primarily by two factors: first, the continuous generation of novel recombinants due to co-infection with multiple HIV-1 strains within populations, some of which establish stable transmission chains; and second, improved molecular surveillance leading to the detection of previously unrecognized CRFs that emerged earlier. Globally, CRF02_AG (33.90%), CRF01_AE (23.00%), and unique recombinant forms (URFs, 26.70%) represent the most prevalent HIV-1 strains (Hemelaar et al., 2020). In China, the predominant circulating lineages include CRF07_BC, CRF01_AE, CRF08_BC, and subtype B′ (Liu et al., 2023). The widespread co-circulation of CRF01_AE and CRF07_BC has facilitated the emergence of numerous second-generation recombinants, particularly 01/07 CRFs and URFs, with MSM serving as the key host population. Of the 20 reported 01/07 CRFs in China to date, at least 17 were first identified within MSM.

Hangzhou, as the economically dynamic capital of Zhejiang Province and a key city in the Yangtze River Delta region, exhibits high population mobility, with migrants comprising 42.0% of its total population (Zhejiang Bureau of Statistics, 2022). This characteristic makes the city a critical hub for regional HIV transmission (Zhang et al., 2017). Between 2019 and 2023, approximately 64.6% of newly diagnosed HIV cases in Hangzhou were attributed to transmission through MSM contact (Zhu et al., 2024). Recent molecular epidemiological surveillance has revealed complex genetic diversity of HIV-1 within the city, with URFs becoming increasingly prevalent and forming transmission clusters that play a significant role in cross-regional spread (Zhu et al., 2025). Consequently, investigating whether novel CRFs have emerged from URF transmission clusters in Hangzhou has become increasingly urgent. In this study, we identified a novel CRF, designated CRF205_0107, from a large URF transmission cluster in Hangzhou. We analyzed near-full-length genome sequences, constructed a structural model based on recombination breakpoints, and used phylogenetic analysis to infer its parental origins, drug resistance profile, and coreceptor tropism, thereby providing a scientific basis for HIV prevention and control strategies in the region.

## Materials and methods

### Study subjects

Plasma samples were collected from treatment-naïve individuals infected with HIV-1 in Hangzhou between 2019 and 2024 for viral drug resistance testing and molecular epidemiological surveillance. From a total of 5,960 partial *pol* gene sequences (HXB2 reference positions: 2253–3,306 nt), 42 sequences formed an independent monophyletic transmission cluster, designated C_1 (Supplementary Figure S1), which was phylogenetically distinct from all known CRFs. Plasma samples from four individuals within this cluster with no known epidemiological links (20HZ2132, 20HZ2416, 23HZ2057, and 24HZ0427) were obtained for comprehensive virological characterization via NFLG amplification and sequencing to identify a potential novel CRF. This study was approved by the Ethics Review Committee of the Hangzhou Center for Disease Control and Prevention, and written informed consent was obtained from all participants.

### Near-full-length genome amplification, sequencing, and assembly

Viral RNA was extracted from 140 μL of plasma using the QIAamp Viral RNA Mini Kit (Qiagen, Valencia, CA, USA). cDNA was synthesized using the SuperScript III First-Strand Synthesis System (Invitrogen, USA) with two sets of overlapping primers (Xing et al., 2021). Two overlapping HIV-1 half-genome fragments were subsequently amplified by nested PCR using ExTaq polymerase (TaKaRa, Dalian, China). Positive PCR products were purified and sent for direct sequencing by a commercial company (Hangzhou Qingke Biotechnology Co., Ltd.). NFLGs were assembled and edited using Sequencher V5.1 (Gene Codes, USA).

### Phylogenetic analysis

The four NFLGs were initially aligned with the HXB2 reference sequence using MAFFT v7.5 (Katoh and Standley, 2013) and manually adjusted in BioEdit v7.2.5 (Hall, 1999). The HIV BLAST tool from the Los Alamos HIV Sequence Database^2^ was used to search for highly similar sequences. A sequence from Hebei Province, S114 (accession: OP921950), showed 98% genetic similarity to our four sequences. Reference sequences encompassing 10 group M subtypes (A–D, F–H, J, L) and CRFs (01_AE, 07_BC, 08_BC, 55_01B) were downloaded from the Los Alamos database. These, along with our four NFLGs and S114, were aligned. Maximum-likelihood (ML) tree was constructed from the alignment using IQ-TREE v2.0 (Nguyen et al., 2015) under the General Time Reversible model with a Gamma distribution rate heterogeneity (GTR + G). Branch support was assessed using the Shimodaira-Hasegawa approximate likelihood ratio test (SH-aLRT), with values ≥90% considered significant (Guindon et al., 2010). The ML tree was visualized using the Interactive Tree of Life online tool iTOL v6^3^.

### Recombination breakpoint analysis

Recombination in the five NFLGs was initially detected using the recombination identification program (RIP) (Siepel et al., 1995) and the jumping profile Hidden Markov Model program (jpHMM) (Zhang et al., 2006). The mosaic structure was further analyzed by bootscanning (Salminen et al., 1995) in SimPlot v1.3.5 (Samson et al., 2022), with putative parental references (CRF01_AE and CRF07_BC) and an outgroup reference (subtype L, AF457101) selected based on similarity plots. Bootscanning was performed with a window size of 350 nucleotides and a step size of 20 nucleotides, using the neighbor-joining method based on the Kimura two-parameter model. To confirm inter-subtype recombination breakpoints, ML trees were constructed for each subgenomic region using the same phylogenetic approach. Each fragment alignment included reference sequences of relevant HIV-1 group-M subtypes, supplemented with Chinese CRF01_AE sub-clusters (C1-C11) and CRF07_BC sub-clusters (N and O) as parental subtype references (Wang et al., 2024). All sub-genomic trees were visualized in iTOL v6.0. The final genomic structure of the recombinant was illustrated using the Recombinant HIV-1 Drawing Tool^4^.

### Time of origin estimation

To elucidate the origins of the different parental lineages within CRF205_0107, Bayesian coalescent Markov Chain Monte Carlo (MCMC) analysis was performed using BEAST v1.10.4 (Suchard et al., 2018). This analysis used the concatenated gene fragments to estimate the time of the most recent common ancestor (tMRCA) for this newly defined CRF. Prior to BEAST analysis, TempEst v1.5.3 (Rambaut et al., 2016) was used to assess the correlation between genetic distance and sampling time to ensure sufficient temporal signal. The analysis employed the GTR + G + I nucleotide substitution model, an uncorrelated log-normal relaxed molecular clock model, and a Bayesian skyline coalescent model (Drummond et al., 2005). MCMC chains were run for 2 × 10^7^ generations, and convergence was assessed using Tracer v1.6 (Rambaut et al., 2018), ensuring that effective sample size (ESS) values of all parameters were >200. After discarding 10% as burn-in, TreeAnnotator was used to generate a maximum clade credibility tree from the posterior tree distribution. Uncertainty for all parameters is reported as the 95% highest posterior density (HPD) interval. The final tree was visualized using FigTree v1.4.4^5^.

### Coreceptor tropism analysis

HIV-1 coreceptor usage was predicted using the Geno2pheno v3.4 online tool^6^. The false-positive rate (FPR) threshold was set at 10% to distinguish viral phenotypes: sequences with an FPR ≤ 10% were classified as CXCR4-tropic, and those with an FPR > 10% as CCR5-tropic (Vandekerckhove et al., 2011).

## Results

### Characteristics of study samples

Individuals in the C_1 cluster were diagnosed with HIV-1 infection between 2019 and 2024. All were male, with the majority (88.1%, 37/42) reporting infection through MSM contact and the remainder through heterosexual contact. The mean age was 32.3 ± 10.1 years. Most cases (76.2%, 32/42) were residents of Hangzhou City. The remaining individuals resided in Anhui Province (*n* = 1), Henan Province (*n* = 1), Jilin Province (*n* = 1), Jiangxi Province (*n* = 2), Yunnan Province (*n* = 2), and other cities in Zhejiang Province (*n* = 3) (Supplementary Table S1). Table 1 summarizes the sociodemographic and clinical information for the five individuals with NFLG data (20HZ2132, 20HZ2416, 23HZ2057, 24HZ0427, and S114). The lowest baseline CD4^+^T-cell count was observed in 23HZ2057 (11 cells/μL), with an average of 163 cells/μL. The average baseline viral load was 1.09E+06 copies/mL.

**Table 1:** Sociodemographic characteristics of five CRF205_0107 infected individuals.

Sequence name	Sampling year	Patient sex	Age	Route	Sample city	Baseline CD4 (cells/μL)	Baseline VL (copies/mL)	Sequence length (based on HXB2)	Accession number
20HZ2132	2020	Male	<30	MSM	Hangzhou	162	6.93E+05	9,102 (634–9,719)	PX262425
20HZ2416	2020	Male	41-50	MSM	Hangzhou	307	3.51E+06	9,105 (634–9,719)	PX262426
23HZ2057	2023	Male	41-50	MSM	Hangzhou	11	1.67E+05	9,081 (634–9,719)	PX262428
24HZ0427	2024	Male	<30	MSM	Hangzhou	172	1.37E+04	9,075 (634–9,719)	PX262427
S114	2021	Male	41-50	MSM	Hebei	—	—	8,974 (633–9,613)	OP921950

### NFLG sequence phylogenetic analysis

**Figure 1:**
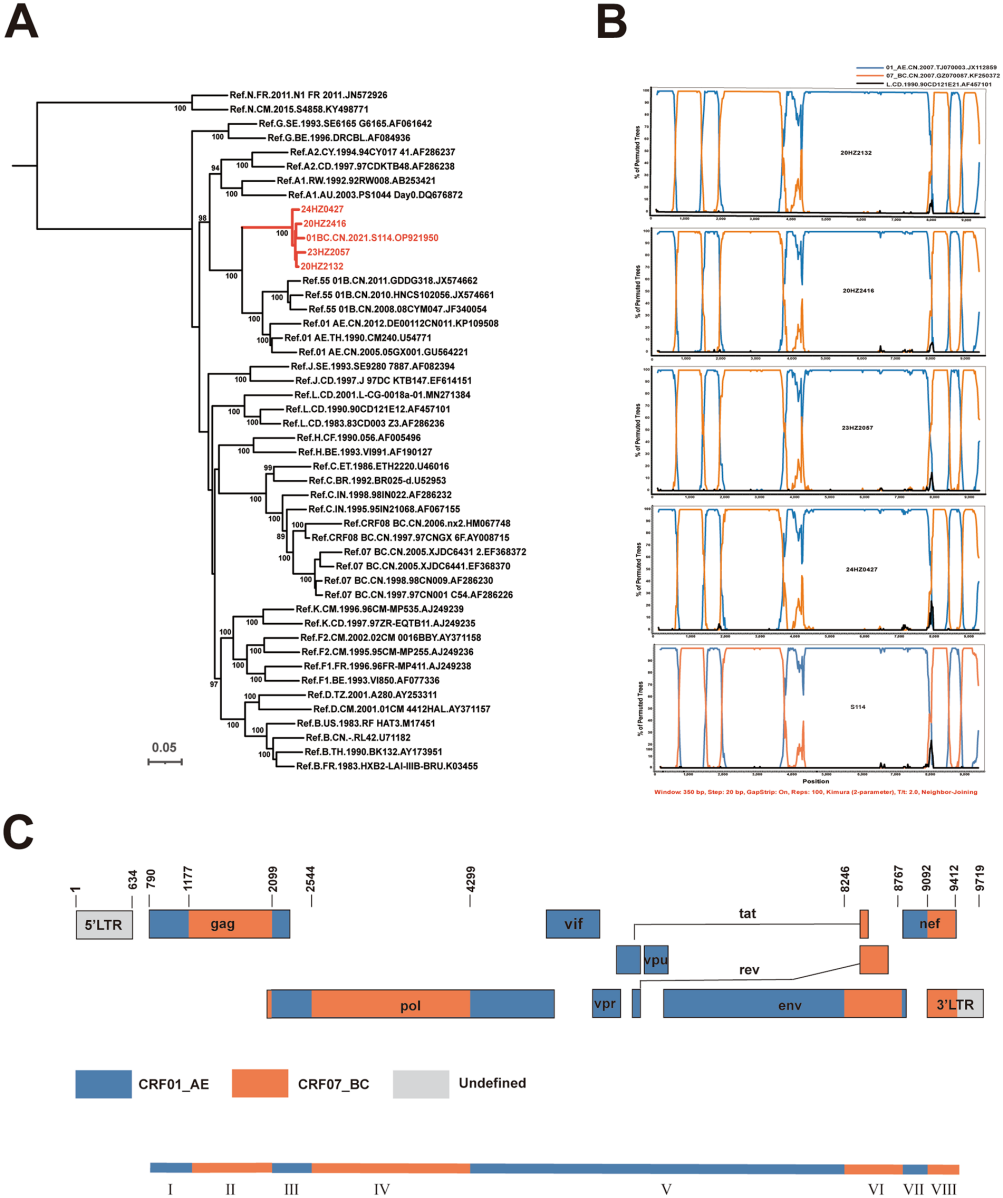
Phylogenetic and recombination analyses of CRF205_0107. **(A)** The ML tree was constructed using general time reversible (GTR) substitution model with the ultrafast bootstrap value 1,000 to assess the reliability of internal branches in IQ-Tree softwarev2.0. The reference sequences were downloaded from the Los Alamos National Library (LANL) HIV database (https://www.hiv.lanl.gov/), including subtypes A–D, F–H, J, K, L, CRF01_AE, CRF07_BC, CRF08_BC, and CRF55_01B together with CRF205_0107 sequences. The sequences of CRF205_0107 were marked with red branch. More than 85% of the bootstrap values were categorized as reliable clusters and labeled at the nodes. **(B)** Bootscan analysis was conducted using a window size of 350 bp and a step size of 20 bp along with reference strains of CRF07_BC, CRF01_AE, and a representative HIV-1 L group sequence. **(C)** The genomic maps of CRF205_0107. The genome mosaic map of CRF205_0107 was drawn according to the LANL Recombinant HIV-1. Drawing Tool (https://www.hiv.lanl.gov/content/sequence/DRAW_CRF/recom_mapper.html).

The NFLGs obtained from samples 20HZ2132, 20HZ2416, 23HZ2057, and 24HZ0427 were 9,102, 9,105, 9,075, and 9,081 nt in length, respectively, spanning from the 5′ LTR to the 3′ LTR, corresponding to the location 634–9,719 nt of HXB2 strain. The previously reported sequence S114 from Hebei Province was 8,974 nt, covering HXB2 positions 633–9,613 nt. The ML tree demonstrated that these five sequences formed a tight monophyletic cluster with 100% bootstrap support and were distinctly separate from other known HIV-1 subtypes and CRFs (Figure 1A), indicating the likely circulation of a novel CRF in Hangzhou.

### NFLG sequence recombination breakpoint and subregional phylogenetic tree analysis

Analysis of the five NFLGs using RIP and jpHMM confirmed recombination between the CRF01_AE and CRF07_BC lineages. Similarly, bootscanning and similarity plot analyses revealed that all five NFLGs shared a highly similar recombination pattern, with the full genome divided into eight segments by seven breakpoints (Figure 1B). Using the HIV-1 Recombinant Drawing Tool from the Los Alamos database, a schematic representation of the chimeric genome structure of CRF205_0107 was generated (Figure 1C). Based on the recombination analysis and using HXB2 as the reference, the genome was partitioned into the following regions: I (790–1,176 nt), II (1,177–2,098 nt), III (2,099–2,543 nt), IV (2,544–4,228 nt), V (4,229–8,245 nt), VI (8,246–8,766 nt), VII (8,767–9,091 nt), and VIII (9,092–9,412 nt) (Figure 1C).

**Figure 2:**
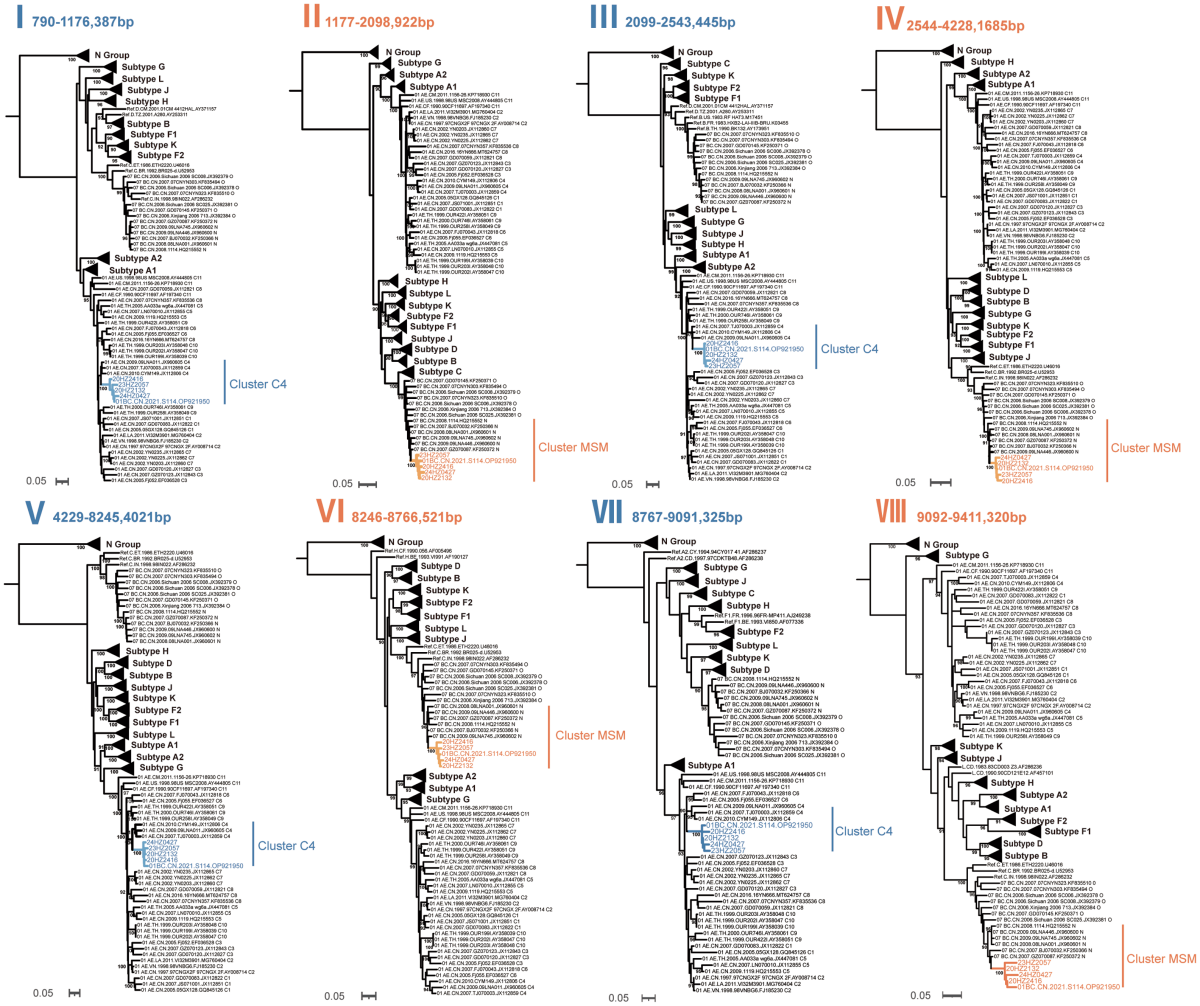
Subregion phylogenetic analysis of CRF205_0107. All the subregion ML trees were reconstructed using the same references, including a group N sequence, twelve subtypes (A1, A2, B–D, F1, F2, G–H, and J–L) and more Chinese CRF01_AE plus more Chinese CRF07_BC. The subregion ML trees were constructed using general time reversible (GTR) substitution model with the ultrafast bootstrap value 1,000 to assess the reliability of internal branches in IQ-Tree software v2.0. More than 85% of the bootstrap values were categorized as reliable clusters and labeled at the nodes. Subregion phylogenetic analyses indicated that the segment I, III, V, and VII of CRF205_0107 belonged to the CRF01_AE_C4. The segment II, IV, VI, and VIII of CRF205_0107 were clustered with the CRF07_BC_N cluster.

To further confirm recombination breakpoints and investigate potential parental lineages, subregional phylogenetic analyses were performed on the eight genomic fragments. High bootstrap support values in the phylogenetic trees confirmed their close relationships with either CRF01_AE or CRF07_BC subtype reference sequences. The partitioned analysis results indicated that fragments I, III, V, and VII clustered within the C4 sub-branch of CRF01_AE, while fragments II, IV, VI, and VIII clustered within the N sub-branch of CRF07_BC (Figure 2).

These strains met the CRF definition criteria of “≥3 cases with no epidemiological linkage and consistent recombinant structure,” and were subsequently accepted by the Los Alamos HIV Sequence Database and officially designated as CRF205_0107.

### Spatiotemporal dynamics analysis

**Figure 3:**
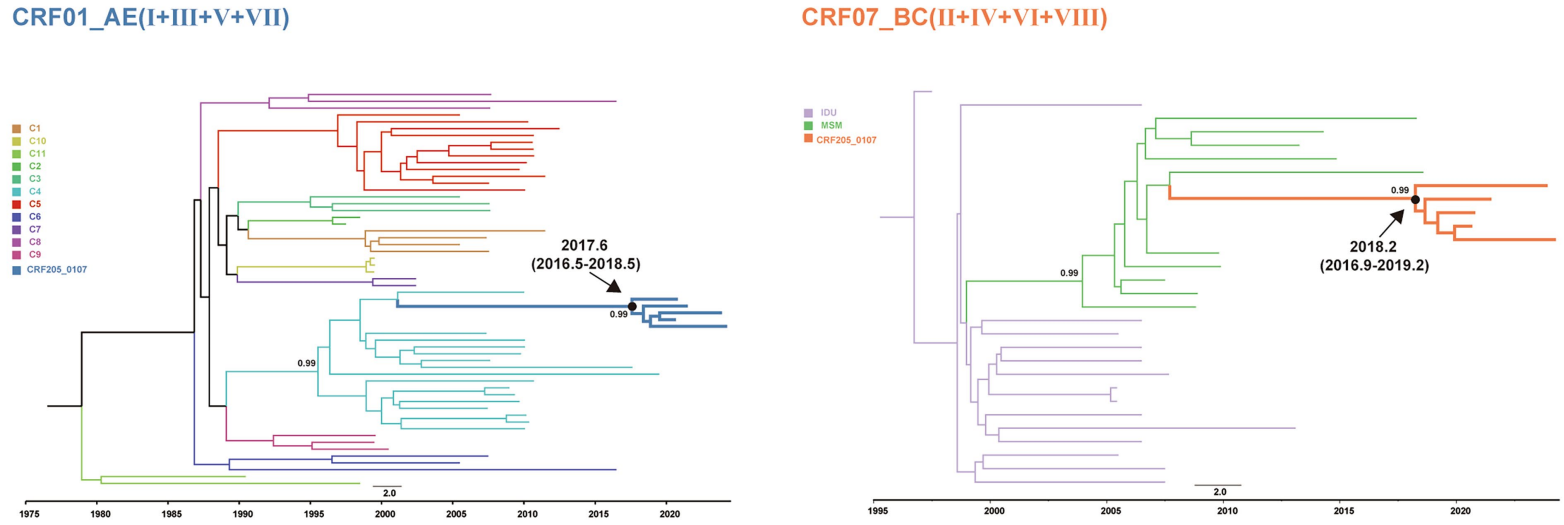
Bayesian phylogenetic analyses were performed using BEAST software v1.10.4. The MCC trees for CRF01_AE and CRF07_BC regions of CRF205_0107 were visualized and edited in FigTree v1.4.0. The mean tMRCA and 95% HPD for the key nodes were displayed in MCC trees.

Bayesian phylogenetic analysis based on the CRF01_AE associated fragments (I, III, V, and VII) and the CRF07_BC associated fragments (II, IV, VI, and VIII) estimated the time to the most recent common ancestor (tMRCA), the tMRCA for the CRF01_AE was 2017.6 [95% HPD: 2016.5, 2018.5], while the tMRCA for the CRF07_BC was 2018.2 (95% HPD: 2016.9, 2019.2). (Figure 3). Consequently, HIV-1 CRF205_0107 is inferred to have originated approximately between 2017 and 2018. The evolutionary analysis also showed that the CRF01_AE concatenated segments clustered with the 01_AE_C4, while the CRF07_BC concatenated segments clustered with 07_BC_N.

### Coreceptor tropism prediction

HIV-1 coreceptor tropism prediction based on the amino acid sequences of the envelope gp120 V3 loop revealed that all five NFLGs contained V3 loops of 35 residues, featuring a highly conserved crown motif (GPGQ) at the tip, which is considered a focal point for potent neutralizing antibody epitopes. The false-positive rate (FPR) values for 20HZ2132, 20HZ2416, 23HZ2057, 24HZ0427, and S114 were 3.7%, 1.7%, 8.6%, 6.9%, and 6.7% (Supplementary Table S2), respectively. Applying the 10% FPR cutoff threshold, all were predicted to be CXCR4-tropic.

## Discussion

CRF205_0107 was identified through molecular surveillance in Hangzhou, originating from a major transmission cluster (C_1) predominantly involving MSM. Cases within this cluster were diagnosed between 2019 and 2024, with geographic distribution covering five provinces beyond Zhejiang, spanning both northern and southern China. Cluster C_1 was previously reported in our research on the spatiotemporal transmission patterns of HIV-1 in large migration hubs as a unique recombinant form (URF, CRF07_BC/CRF01_AE) cluster (HZC1), which demonstrated significant cross-regional transmission capacity, playing a role in key transmission pathways between Hangzhou and other economically developed regions like Beijing and Shenzhen (Zhu et al., 2025). These findings indicate that the novel recombinant strain CRF205_0107 has established sustained transmission within the MSM population and is disseminating in conjunction with population mobility.

The distribution of HIV-1 genotypes exhibits distinct population and geographic specificity. The regional spread of specific variants is often attributed to unique viral characteristics and transmission opportunities through specific pathways. For example, CRF55_01B was first identified in the MSM population in Shenzhen, Guangdong Province (Han et al., 2013), and subsequently spread rapidly to heterosexual populations. A nationally representative study on the molecular evolutionary history of viral sequences indicated that CRF55_01B likely emerged around 2003, underwent rapid expansion between 2005 and 2009, and has continued to circulate since 2010 (Gan et al., 2021). The high mobility of MSM populations combined with the rapid development of transportation networks jointly facilitated the widespread northward and inland spread of CRF55_01B from coastal areas. To date, it has become the fifth most prevalent HIV-1 subtype in China and has been detected in all provinces. CRF205_0107, similarly identified in MSM, emerged around 2017–2018. As a comparatively “younger” strain, it has already formed a transmission cluster in a specific region and population. It is therefore necessary to conduct nationwide subtype surveillance for CRF205_0107 to promptly identify its transmission dynamics and prevent its national spread through MSM populations and the convenient transportation network centered in Hangzhou, which holds significant implications for HIV-1 prevention and control efforts.

The co-circulation of CRF01_AE and CRF07_BC within MSM populations provides ample opportunity for the generation of recombinant viruses. CRF205_0107, which originated from these two parental strains, contains seven recombination breakpoints. The tMRCA for the CRF01_AE (2017.6) preceded that of the CRF07_BC (2018.2), suggesting at least two recombination events occurred between 2017 and 2018. Tracing the origins of parental strains for novel recombinants holds significant scientific value for elucidating associations between different transmission routes and viral subtypes, as well as for revealing potential characteristics of “bridge populations.” CRF01_AE, originating in Africa, was the first successfully circulating HIV-1 recombinant and has spread globally, particularly in Southeast and East Asia. Currently, CRF01_AE strains are further divided into 11 clusters, exhibiting high diversity and rapid spread; among these, clusters 4 and 5 are highly prevalent among MSM in northern China (Wang et al., 2024). CRF07_BC, the first recombinant virus identified in China, is widely believed to have originated among injection drug users (IDUs) in Yunnan Province (Feng et al., 2016) and subsequently spread to other regions of China, especially Beijing, Shanghai, Guangdong, and Zhejiang (Vrancken et al., 2020). During its spread, CRF07_BC differentiated into two clusters: 07BC_O and 07BC_N. 07BC_O represents the original CRF07_BC, primarily circulating among IDUs and heterosexual populations, concentrated in southwestern and northwestern Chinese provinces. 07BC_N is a newer cluster, mainly found among MSM in northern Chinese provinces (Ge et al., 2021), driven by expansion within northern MSM networks and facilitating “north-to-east” spread (Zhu et al., 2025). Fragment evolutionary analysis revealed that the CRF01_AE and CRF07_BC fragments within CRF205_0107 clustered with the 01_AE_C4 and 07BC_N variants prevalent among MSM in northern China, respectively. Furthermore, three other novel CRFs (CRF158_0107, CRF168_0107, and CRF170_0107) recently reported, are also formed by recombination between 01_AE_C4 and 07_BC_N (Chen et al., 2024; Xiao et al., 2025; Li et al., 2024). The co-circulation of multiple strains within the same geographic region and among high-risk populations leads to co-infection or super-infection, thereby fostering novel recombinants. The high-frequency recombination between 01_AE_C4 and 07_BC_N underscores the necessity for close monitoring of viral transmission and evolution across different regions and risk groups.

Retroviruses can rapidly adapt to their environment, utilizing error-prone enzymes for genetic and phenotypic evolution to optimize survival strategies and potentially enhance virulence (Swanstrom and Coffin, 2012). Numerous studies have observed that individuals infected with CRF01_AE strains exhibit a higher proportion of CXCR4 tropism compared to those infected with non-CRF01_AE strains (Oon et al., 2011; Li Y. et al., 2014; Li X. et al., 2014). Focusing on MSM cohorts, researchers observed significantly lower CD4 counts in 01_AE_C4 compared to 01_AE_C5, with 01_AE_C4 infection associated with faster CD4 decline and shorter overall survival (Li Y. et al., 2014; Li X. et al., 2014). In this study, the newly identified circulating recombinant form CRF205_0107 contains an *env* gp120 region (HXB2:6225–7,758 nt) belonging to 01_AE_C4. V3 loop-based coreceptor usage prediction indicated all five viruses were CXCR4-tropic, suggesting that CRF205_0107 may possess a high propensity for CXCR4 tropism. However, this finding is based on only five samples and has not been phenotypically validated. Furthermore, given that the parental lineage 01_AE_C4 itself has a high proportion of X4-tropic strains, the observed tropism may also be lineage-determined. Therefore, the CXCR4 tropism of CRF205_0107 needs to be further investigated in larger cohorts.

Furthermore, this indicates that caution should be exercised when considering CCR5 coreceptor antagonist therapy for individuals infected with the CRF205_0107 strain.

There are two limitations in this study. The first is the relatively small sample size. Due to limitations in sample quality and the exclusion of epidemiologically linked cases, we obtained only four near-full-length genome sequences from cluster C_1. The second limitation is that the samples were collected between 2019 and 2024 exclusively in Hangzhou, which may not reflect the current prevalence pattern of the CRF205_0107 strain in the broader population. Whether this novel CRF has caused a widespread epidemic in surrounding areas or even nationwide among the MSM population and other high-risk groups requires continued attention.

## Conclusion

In conclusion, we identified a novel HIV-1 CRF, designated CRF205_0107, for the first time in Hangzhou, Zhejiang Province. The emergence of CRF205_0107 demonstrates that recombination between CRF01_AE and CRF07_BC is continuously occurring and evolving, further increasing the complexity of HIV-1 genetic diversity in China. It highlights the important role of the MSM population in the emergence and spread of novel HIV-1 recombinants and emphasizes the urgent necessity for sustained, real-time surveillance of HIV-1 recombination events within this key population in specific regions. Blocking the cross-regional transmission of novel recombinant forms will undoubtedly have a profound impact on epidemic control.

## Data Availability

The datasets presented in this study can be found in online repositories. The names of the repository/repositories and accession number(s) can be found in the article/Supplementary material.

## Footnotes

1
https://www.hiv.lanl.gov/content/sequence/HIV/mainpage.html

2
https://www.hiv.lanl.gov/

3
https://itol.embl.de

4
www.hiv.lanl.gov/content/sequence/DRAW_CRF/recom_mapper.html

5
http://tree.bio.ed.ac.uk/software/figtree/

6
https://www.geno2pheno.org/
