# Supplementary figures and images for: Molecular genetic characterization of a novel HIV-1 circulating recombinant form (CRF205_0107) among men who have sex with men in Hangzhou, China
Source: Front Microbiol. 2026 Apr 9;17:1794333. doi: 10.3389/fmicb.2026.1794333 (PMC13102639; doi:10.3389/fmicb.2026.1794333)

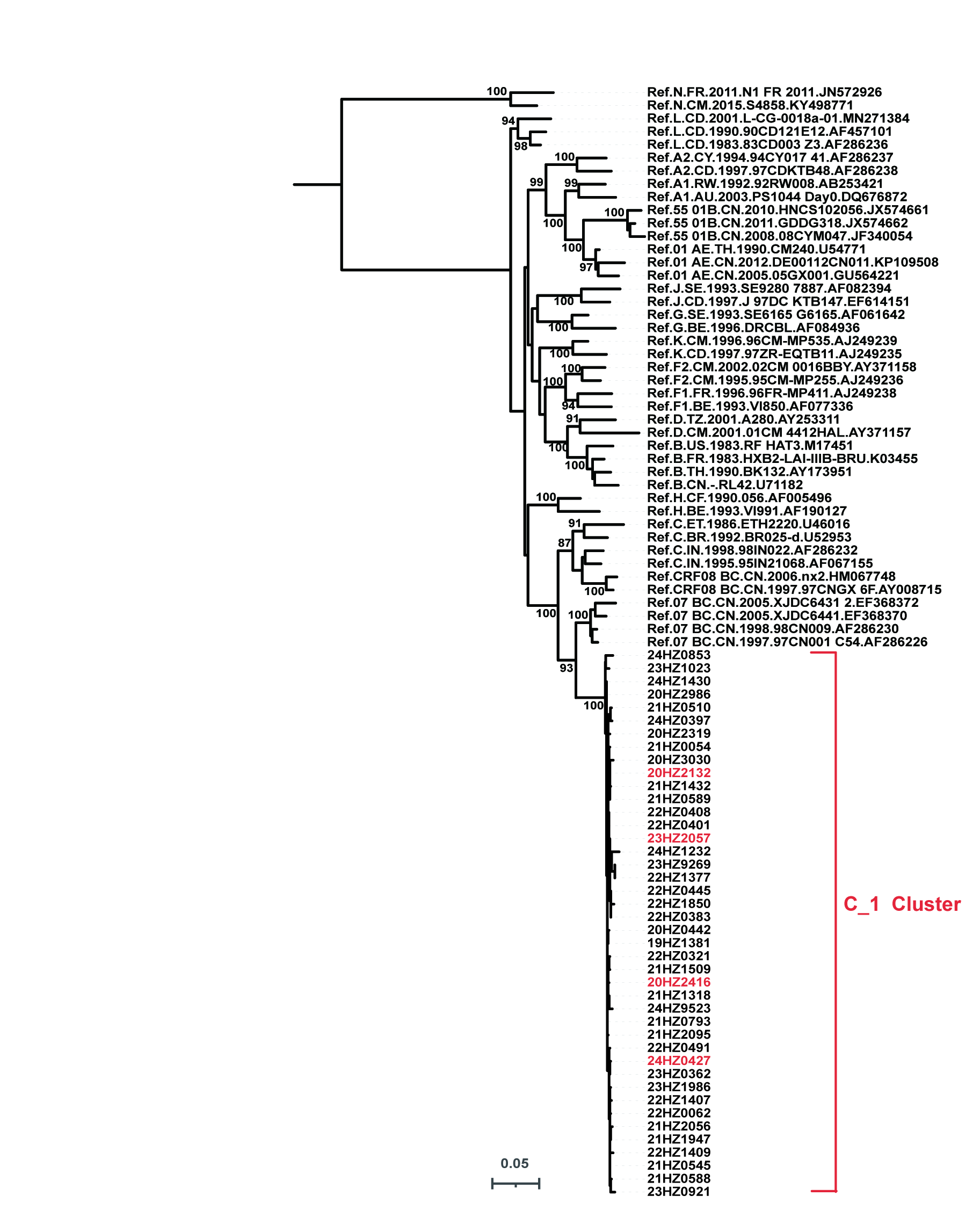

Supplement: Supplementary file 1 [file Image_1.tif]
