# Supplementary material for: Molecular genetic characterization of a novel HIV-1 circulating recombinant form (CRF205_0107) among men who have sex with men in Hangzhou, China
Source: Front Microbiol. 2026 Apr 9;17:1794333. doi: 10.3389/fmicb.2026.1794333 (PMC13102639; doi:10.3389/fmicb.2026.1794333)
Supplement: Supplementary file 2 [file Table_1.docx]

| Supplementary Table S1 Demographic characteristics of C_1 cluster(n = 42) | | |
| --- | --- | --- |
| Characteristic | | Patients |
| Sex | Female | 0 |
|  | Male | 42 |
| Risk factor | HET | 5 |
|  | MSM | 37 |
| Age | ≤ 30 | 21 |
|  | 31-40 | 12 |
|  | 41-50 | 7 |
|  | ≥ 51 | 2 |
| Location | Hangzhou, Zhejiang Province | 32 |
|  | Anhui Province | 1 |
|  | Henan Province | 1 |
|  | Yunan Province | 2 |
|  | Jilin Province | 1 |
|  | Jiangxi Province | 2 |
|  | Taizhou, Zhejiang Province | 2 |
|  | Huzhou, Zhejiang Province | 1 |
| Sample year | 2019 | 1 |
|  | 2020 | 6 |
|  | 2021 | 12 |
|  | 2022 | 11 |
|  | 2023 | 6 |
|  | 2024 | 6 |

NOTE：

MSM: Men who have Sex with Men

HET: heterosexual

| Supplementary Table S2 Cell tropism prediction based on the amino acid sequence of the CRF205_0107 V3 loop | | | | |
| --- | --- | --- | --- | --- |
| Sequence  name | V3-LOOP^1^ | Length | Subtype | FPR^2^ |
| 20HZ2132 | CTRPFNNTRTSVRIGPGQVFYRTGEIIGDIRKAYC | 35 | AE | 3.7 |
| 20HZ2416 | CTRPFNNTRTSTRIGPGQVFYRTGEIIGDIRKAYC | 35 | AE | 1.7 |
| 24HZ0427 | CTRPSNNTRTSVRIGPGQVFYRTGEIIGDIRKAYC | 35 | AE | 6.9 |
| 23HZ2057 | CTRPSNNTRTSVRIGPGQVFYRTGDIIGDIRKAYC | 35 | AE | 8.6 |
| S114 | CTRPFNNTRESVRIGPGQVFYRTGEIIGDIRKAYC | 35 | AE | 6.7 |

NOTE：

1Residues with a grey background showed the crown motif (amino acids 15-18) in the tip of V3 region, which was considered as the focal point of the potent neutralising antibody epitope

2FPR: false positive rate, the probability of classifying an R5-virus falsely as X4

Supplementary Figure S1

ML tree of *pol* sequences(HXB2 reference positions: 2253–3306 nt) of the C_1 cluster. Sequences obtained by us are in red.Only bootstrap values ≥ 85% are shown.
